# New format, Open Access, and online pre-publication

**DOI:** 10.1080/17453670902804406

**Published:** 2009-02-01

**Authors:** Anders Rydholm, Olle Svensson

As you have perhaps just noted, Acta Orthopaedica has changed format to the one used by most journals today. The decision to abandon the smaller format, used since the start of Acta in 1930, was not easy; it was, in fact a rather emotional one. The editorial board and most readers really liked the small, handy format—designed to fit into the pocket of a white coat. In a way, it symbolized Acta's ethos. The main reason for changing was, alas, economic.

We need to lower our production costs because of Open Access, which we started in 2005. This means that all our articles are accessible as full text—for everyone, at no cost—via PubMed immediately after the printed version has been published. We do not use the common system of delayed Open Access with articles that first become freely available 6–12 months after publication or are free to subscribers only. This, in turn, means that one important source of income, library subscriptions, will diminish. Furthermore, all articles published since the start in 1930 are free to everyone on Acta's website at www.actaorthop.org

Open Access, by definition free of charge to users, has to be paid for by someone: “there ain't no such thing as a free lunch”. Many journals have introduced page charges for authors to cover the costs. Acta Orthopaedica has decided that Open Access should also be without costs for authors, which means that we have to cut production costs. At present we are breaking even, but in a couple of years, unless we find other sources of income, Acta will perhaps also have to introduce page charges, to compensate for increasing general costs, unchanged subscription fees for members, and the reduced number of library subscriptions that will come in the foreseeable future. A drastic way of cutting costs would be to stop printing Acta on paper. However, we believe that most readers (and all editors) still love the smell and feel of paper and papers.

The time between submission and acceptance has been reduced. Four-fifths of all submitting authors get an answer within 6 weeks. For the circa 20% manuscripts accepted after peer review, most are ready for publishing within 3–4 months (depending on how fast the authors correspond to the revisions, re-revisions, statistical and linguistic issues, picture and table editing, and so on). The mean time between acceptance and publication on paper is around 6 months. We try to reduce it, but since we have a rather lean production line, we do not foresee a major reduction—and this is perhaps not even desirable. One reason is that we try to publish articles on the same subject together in the same issue, often accompanied by an Editorial. This means that some of the articles have to wait until the latest one to be submitted is ready for publication. To reduce the time between acceptance and publication, like many other journals Acta will now start online pre-publication. This means that the article will be available through Open Access on PubMed (“Epub ahead of print”) almost immediately after the author proofs have been corrected. Thus, the time between acceptance/electronic publishing and print publication will be of less importance.

If you have any opinions on the future development of Acta Orthopaedica, or on other issues for that matter, please e-mail us at acta.ort@med.lu.se! After all, we are a non-profit organization, owned by the NOF members: our purpose is to spread knowledge—not wealth.

